# P-350. Prescription of Weight Loss Medications in HIV Patients at a Rural Federally Funded Clinic: A Retrospective Study

**DOI:** 10.1093/ofid/ofaf695.568

**Published:** 2026-01-11

**Authors:** Diviya Bharathi Ravikumar, Jay Patel, Barath Prashanth Sivasubramanian, Neha Nanditha Adepu, Kelly Clark, Uzer Abdulaziz Memon, Karthik Basumani, Heer Pareshbhai Shah, Falaknaaz Mubassirhusen Saiyad, Anusha Endreddy, Krishna Sai Kiran Sakalabaktula, Dency Dineshbhai Mavani, Mathangi Murali, Naveen Yellappa, Rutul Dalal, Raghavendra Tirupathi

**Affiliations:** ESIC Medical College and Postgraduate Institute of Medical Science and Research, Chennai, Tamil Nadu, India; B.J. Medical College, Ahmedabad, Gujarat, India; University of Texas Health San Antonio, San Antonio, Texas; Osmania Medical College, Hyderabad, Telangana, India; Keystone Health, Chambersburg, Pennsylvania; Smt. NHL Municipal Medical College, Ahmedabad, Gujarat, India; ESIC Medical College and Postgraduate Institute of Medical Science and Research, Chennai, Tamil Nadu, India; Smt. NHL Municipal Medical College, Ahmedabad, Gujarat, India; Smt. NHL Municipal Medical College, Ahmedabad, Gujarat, India; Alluri Sitarama Raju Academy of Medical Sciences, Eluru, Andhra Pradesh, India; Government General Hospital, Kakinada, Kakinada, Andhra Pradesh, India; Smt. NHL Municipal Medical College, Ahmedabad, Gujarat, India; Government Erode Medical College and Hospital, Chennai, Tamil Nadu, India; Geisinger Commonwealth School of Medicine, Scranton, Pennsylvania; Medical Director, Penn State Health Eastern Region, Penn State Health St. Joseph Medical Center, Pennsylvania, USA, Lancaster, Pennsylvania; Keystone Health, Chambersburg, Pennsylvania

## Abstract

**Background:**

Obesity in people with HIV (PWH) has increased from 13.4% in 2014 to 21.5% in 2020. The usage of weight loss medications (WM) has expanded, and their potential in PWH remains underexplored. This study aimed to evaluate the factors affecting WM prescription in PWH at a non-academic Ryan White clinic in rural Pennsylvania.

**Figure:**
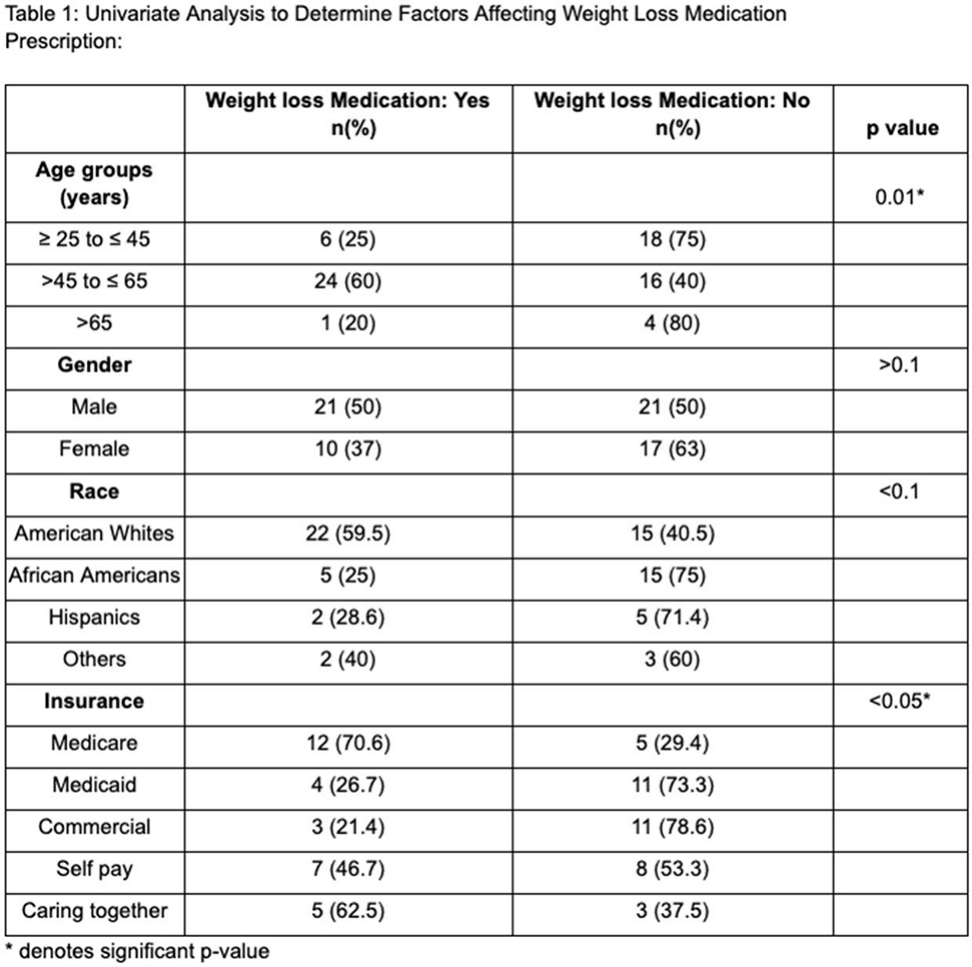

**Figure:**
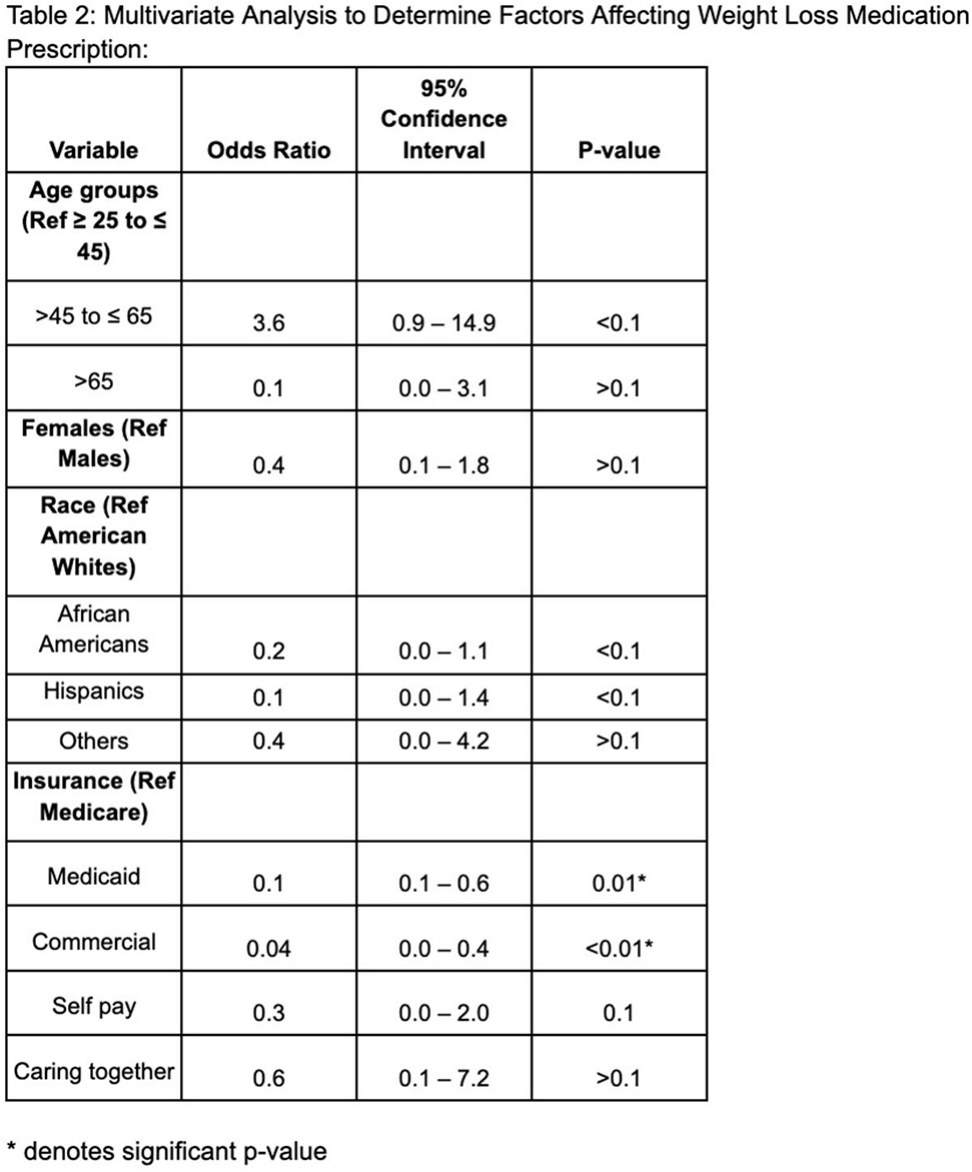

**Methods:**

A retrospective study including PWH on antiretroviral therapy with a BMI ≥ 25, and with at least 1 month of follow-up, was included. In this cohort, 88.4% received integrase-based triple therapy. Descriptive statistics and Fisher’s exact test were performed. Multivariate regression was conducted to identify factors. A p-value ≤ 0.05 was adopted.

**Results:**

Of 69 patients included, 44.9% were on WM. In this, 14.5% were on semaglutide, 7.3% tirzepatide, and 23.2% bupropion. In this cohort, middle-aged patients (45-65 years) were higher than in younger patients (25-45 years), 60% vs 25%, p ≤ 0.01. Males were more than females (50% vs 37%, p > 0.1), and African Americans (AA) were less than American Whites (AW) to be on WM (25% vs 59.5%, p ≤ 0.01). Medicare patients were higher than those with Medicaid or commercial insurance (70.6% vs 26.7% vs 21.4%, p ≤ 0.05). Patients with hypertension, hyperlipidemia, or diabetes were more often on WM than those without (63.3% vs 30.8%, p < 0.01; 72.2% vs 35.3%, p = 0.01; 50% vs 43.6%, p > 0.1). The averages of BMI, weight (lbs), and HbA1c (SI) before treatment were 34.2 ± SE 1.3, 215.6 ± SE 11.1, and 6.6 ± SE 0.4, respectively. The mean BMI difference, mean weight change, and HbA1c change after WM were 0.2 ± SE 0.5, 0.7 ± SE 2.7, and -0.3 ± SE -0.4, respectively. Multivariate regression revealed that middle-aged patients had higher odds of being on WM than younger patients (OR 3.6, 95% CI 0.9-14.9, p < 0.1). AA had decreased odds of being on WM than AW (OR 0.2, p < 0.1). Patients with Medicaid (OR 0.1, 95% CI 0.0-0.6, p = 0.01) and commercial insurance (OR 0.04, 95% CI 0.0-0.4, p < 0.01) were less likely to receive WM than Medicare patients.

**Conclusion:**

Our study revealed that race and insurance play a significant role in access to weight loss management in HIV patients. These disparities affect access to these medications in rural centers. Long-term studies are needed to evaluate the efficacy of weight loss medications in this population.

**Disclosures:**

All Authors: No reported disclosures

